# Frontal Non-Invasive Neurostimulation Modulates Antisaccade Preparation in Non-Human Primates

**DOI:** 10.1371/journal.pone.0038674

**Published:** 2012-06-06

**Authors:** Antoni Valero-Cabre, Nicolas Wattiez, Morgane Monfort, Chantal François, Sophie Rivaud-Péchoux, Bertrand Gaymard, Pierre Pouget

**Affiliations:** 1 Université Pierre et Marie Curie, CNRS UMR 7225, INSERM UMRS 975, Institut du Cerveau et la Möelle (ICM), Paris, France; 2 Laboratory for Cerebral Dynamics Plasticity and Rehabilitation, Boston University School of Medicine, Boston, Massachusetts, United States of America; 3 Cognitive Neuroscience and Information Technology Research Program, Open University of Catalonia (UOC), Barcelona, Spain; CNRS - Université Claude Bernard Lyon 1, France

## Abstract

A combination of oculometric measurements, invasive electrophysiological recordings and microstimulation have proven instrumental to study the role of the Frontal Eye Field (FEF) in saccadic activity. We hereby gauged the ability of a non-invasive neurostimulation technology, Transcranial Magnetic Stimulation (TMS), to causally interfere with frontal activity in two macaque rhesus monkeys trained to perform a saccadic antisaccade task. We show that online single pulse TMS significantly modulated antisaccade latencies. Such effects proved dependent on TMS site (effects on FEF but not on an actively stimulated control site), TMS modality (present under active but not sham TMS on the FEF area), TMS intensity (intensities of at least 40% of the TMS machine maximal output required), TMS timing (more robust for pulses delivered at 150 ms than at 100 post target onset) and visual hemifield (relative latency decreases mainly for ipsilateral AS). Our results demonstrate the feasibility of using TMS to causally modulate antisaccade-associated computations in the non-human primate brain and support the use of this approach in monkeys to study brain function and its non-invasive neuromodulation for exploratory and therapeutic purposes.

## Introduction

Transcranial magnetic stimulation (TMS) has become a widely popular technique to non-invasively interfere with the neuronal activity of a reasonably small volume of tissue in the human brain. It has been shown that single pulses or short TMS bursts can generate *online* interference on ongoing brain processing, whereas longer TMS patterns tailored in frequency and interpulse interval have the potential to induce lasting *offline* effects beyond their own duration. In spite of its widespread use, the underlying neural mechanisms of TMS remain relatively unknown, and the lack of insight on questions such as the intracerebral distribution of magnetically induced electrical currents, their depth, spatial decay and dependency on the state of cortical activity hamper a reliable interpretation of its impact and the further development of this tool for exploratory and therapeutic applications.

Intracortical microstimulation, an invasive homologue technique to TMS, has been widely used in combination with oculometric measurements and mapping techniques, such as electrophysiological and fMRI recordings in non-human primates. Those approaches have provided causal evidence about the role of the Frontal Eye Field’s (FEF), a highly sophisticated cortical area, with direct bearing on oculomotor functions [1], [2]. More recently, they have also revealed the causal contributions of the FEF in attentional orienting [3] and its ability to influence different aspects of visual perception [4], [5]. Likewise, TMS has served in humans to map the FEF’s contributions to saccadic activity [6], [7], [8], [9] and unveil causal relationships between this area and some of the above mentioned processes [10], [11], [12], [13], [14], [15], [16], [17]. Notwithstanding, it has also delineated a role for the human FEF, which is not always easy to reconcile with data provided by FEF microstimulation in monkeys. The most striking one, is that whereas monkey FEF invasive stimulation has the ability to elicit ocular saccades [18], [19], human frontal TMS stimulation in homologue locations, even at high intensities will yield at best slight enlargements of saccadic preparation time, but no signs of eye motion [20], [21]. Some of those discrepancies could reflect differences in the organization of saccadic regions across species. Nonetheless, direct analogies between invasive and non-invasive brain neurostimulation are to be taken with care given the lack of insight on the neural effects induced by the latter.

In an attempt to understand the underlying mechanisms and optimize its applications, TMS has been probed in anesthetized rodents [22], [23], [24], [25] and felines [26], [27], [28], [29], [30] and more recently also in awake freely performing cats [31], [32]. All those studies have greatly contributed to the current understanding of non-invasive neurostimulation effects at different levels of organization. Nonetheless, the small head size of those species as compared to the existing TMS coils, crucial differences in brain structural and functional organization, and the effort required to train such animals in complex cognitive paradigms precludes an efficient comparison and ultimate translation of such findings to humans [33].

In spite of being a plausible alternative to direct cortical microstimulation, TMS has rarely been used in non-human primate models with similar purposes as those of human cognitive neurosciences. Past studies have mainly targeted the monkey primary motor cortex, an area for which an objective electrophysiological output can be quantified. Such approach has been probed in the monkey corticospinal system to assess anesthetics [34], address the local metabolic correlates of M1 rTMS [35], further understand TMS intracortical effects [36], explore motor circuitry in sedated animals [37], map the organization of cortical representations and modulate the excitability of cortico-spinal connectivity [38]. Very recently, high frequency TMS patterns on the frontal cortex have also been reported to induce weak offline interferences in pro-saccadic activity [39]. In spite of all this highly relevant work, an awake non-human primate TMS model, able to induce *online* modulation of a cognitive function as performed in humans, and compatible with an invasive electrophysiological exploration of the neural underpinnings for such effects is yet to be achieved.

We hereby used TMS on the awake macaque frontal cortex to interfere with the activity of antisaccade preparatory processes driven by spatial visual stimuli. We focused in the study of TMS driven interferences on the FEF, a complex area hosting highly overlapped networks likely to be involved in functions such as sensory integration, attentional orienting, oculomotor planning, saccade execution, spatial short term memory, visual detection, discrimination and visual awareness [3], [13], [15], [16], [40], [41]. Our short-term goal was to demonstrate the feasibility of *online* TMS experiments in non-motor areas of the awake and freely performing non-human primate. Such approach used in combination with other mapping methods (such as local field potentials, single unit recordings or fMRI recordings) has the potential to clarify in a near future some of the mechanisms, underlying the effects of TMS, and serve to the causal non-invasive exploration of cognition in non-human primate models.

## Materials and Methods

**Figure 1:**
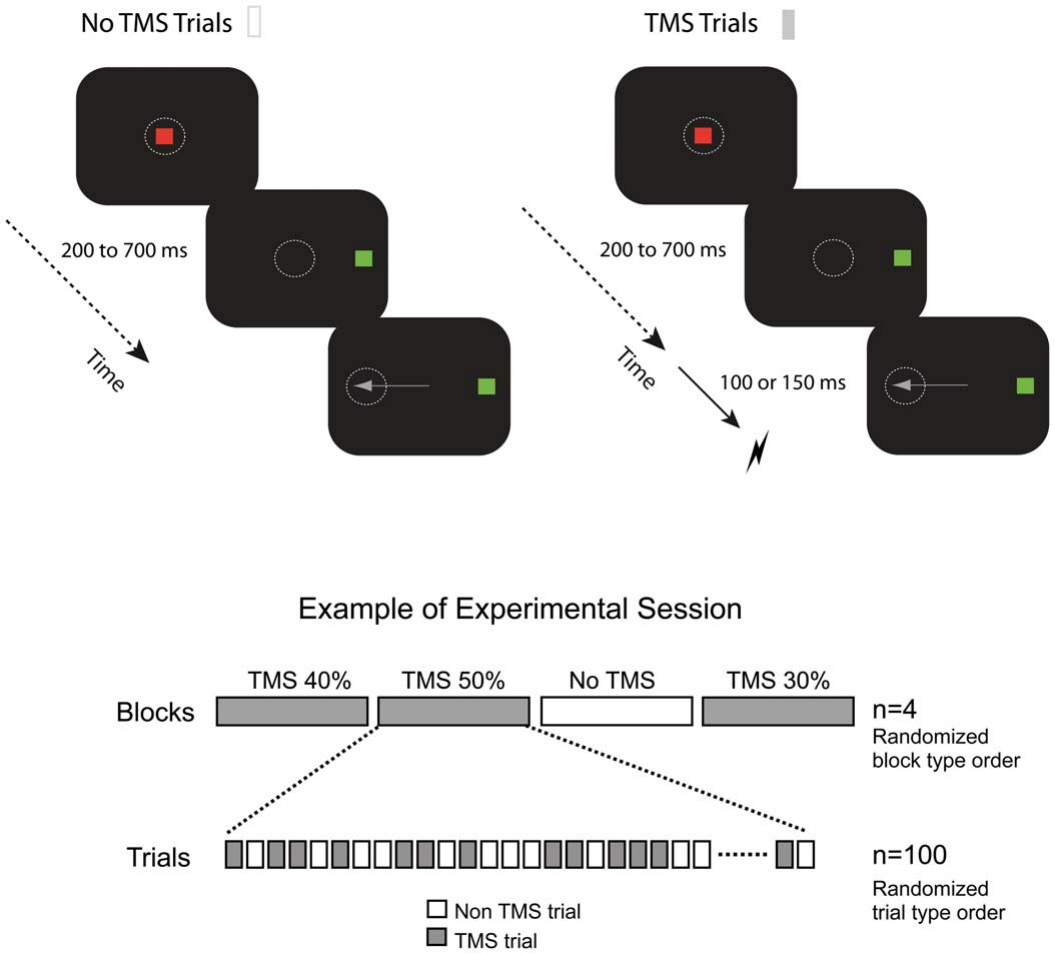
Behavioral paradigm illustrating the experimental antisaccade paradigm. (*Upper panel)* Antisaccade paradigm practiced by the two monkeys under the online impact of sham (left panel) or active (right panel) TMS single pulses. After fixating on a central stimulus (red), monkeys were to initiate a fast saccade to a location in the opposite direction with respect to a peripheral target (green) appearing on the screen, simultaneously (no gap) to the disappearance of the central fixation. Animals performed within each block, no-TMS trials (white small rectangles) yielding no stimulation at all (Upper Left) and TMS trials (grey small rectangles) during which a single TMS pulse was delivered at a given postarget onset SOA prior to the AS initiation, to modulate the planning of visually guided oculomotor activity *(Bottom panel)* Example of an experimental session. Animals performed a total of 4 blocks of AS training per session. In one of the blocks they did not receive TMS (white long rectangle), whereas in the remaining 3, they received in half of the trials TMS pulses (see long grey-filled rectangles) at one of the 3 intensities used in the study (30%, 40% and 50%). The order of the four blocks (3 TMS blocks at 30%, 40% or 50% absolute TMS intensities and 1 noTMS block) was randomized within each session. Monkeys performed 100 trials per block (50 no-TMS and 50 TMS trials) for a total of 400 trials per session, and received 50 pulses per TMS block (i.e., only in 50% of the trials), amounting to 150 pulses per experimental session. Independent sessions comprising active TMS pulses delivered at 100 ms or 150 ms SOA post target onset on the FEF, sham TMS pulses and active TMS stimulation in a control location were carried over.

Two captive-born macaques (one *Maccaca Mulatta*, “Y", and one *Maccaca Fascicularis*, “C") participated in this study. The monkeys were individually housed and handled in strict accordance with the recommendations of the Weatherall Report about good animal practice. Monkey housing conditions, surgical procedures and experimental protocols were all carried out in strict accordance with the National Institutes of Health guidelines (1996) and the recommendations of the EEC (86/609) and the French National Committee (87/848). The authorization for conducting our experiments in the institute was delivered by the Animal Health and Veterinary Medication Division of the Department of Public Veterinary Health, Nutrition and Food Safety of the French Ministry of Health (last renewals. no. *Arrêté prefectoral N° DTPP* 2010-424). Our routine laboratory procedures included an environmental enrichment program where monkeys had access to toys, mirrors and swings. Monkeys also had visual, auditory and olfactory contact with other animals and, when appropriate, could touch/groom each other. Any possible pain associated with surgeries was pharmacologically ameliorated by means of a daily injection of Ketofen (0.03 ml/kg) or Buprecare (0.067 ml/kg). The well-being and health conditions of the monkeys were constantly monitored by an institutional veterinary doctor. Prior to participating in the study, both animals were periodically chaired, head-posted and trained to perform an antisaccade (AS) paradigm (Figure 1, upper panel) for a period of 6–12 months, until they became regular and proficient performers.

### Surgical Procedure

**Figure 2:**
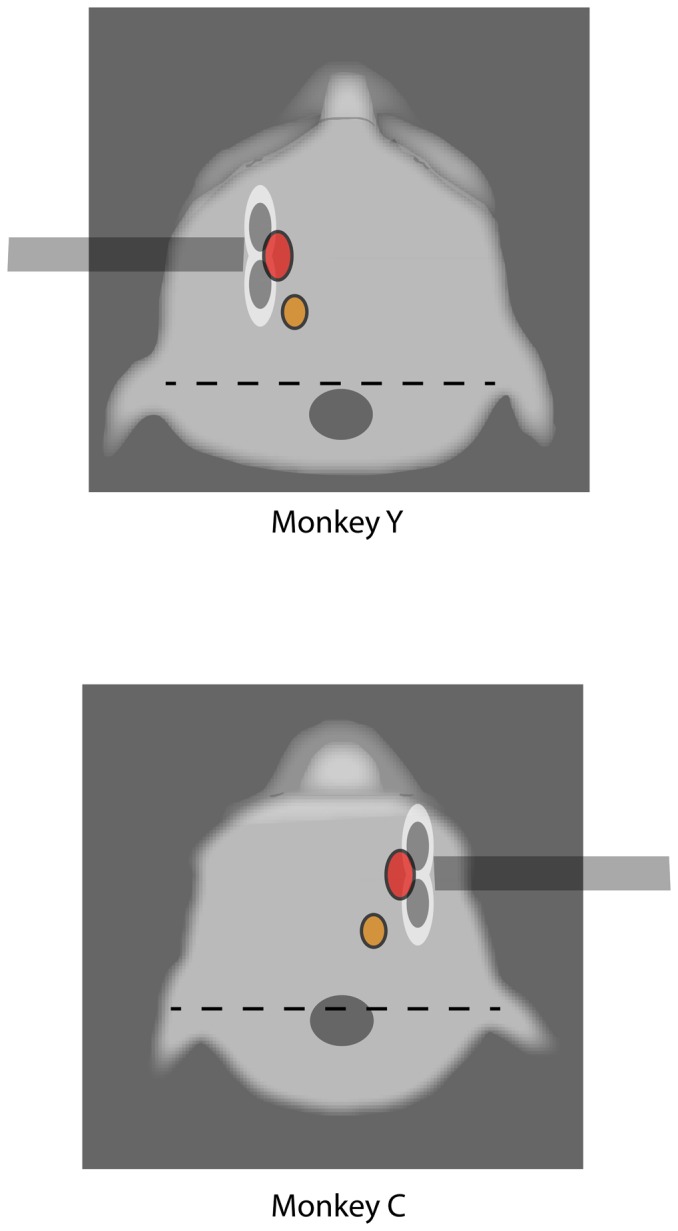
Schematic of TMS sites. Modified picture showing a top view of each of the two monkey’s scalp profiles (animals ‘Y’ and ‘C’), while posted and under training. The dotted line corresponds to the stereotaxic zero bar; the grey dot signals the location and size of the head-post; the orange dot corresponds to the location where digit movements were evoked by TMS pulses; the red dot FEF region of stimulation; the double white/grey dots is an approximate schematic representation of the TMS figure-of-eight coil which was located on the FEF region.

The surgical procedures for titanium headpost implant were the same as previously described [42], [43]. Each animal was deeply anaesthetized with ketamine hydrochloride (5 mg/kg i.m.) for initial sedation and anesthesia was induced with 2–4% isofluoran gas and then maintained during surgery. Heart rate, temperature and respiration were carefully monitored and kept within physiological range. Pain medication was given prior the surgery and routinely given after surgery. Head posts (9/32″ or 7.1 mm internal diameter) were commercially available as Part #6-FHP-X2F produced by Crist Instrument, Hagerstown, MD, USA. They had an “X"-shaped footplate designed for attachment to the skull with a total of 12 titanium bone screws. The vertical post had a tapered cross section, designed to mate with a headpost holder (see Part #6-FHB-S2B, Crist Instrument, Hagerstown, MD, USA). In monkey ‘Y’ the center of the head post was located adjacently caudal to the stereotaxic zero bar, aligned with the interauricular scalp line. In monkey ‘C’ the headpost was placed slightly more rostral than in monkey ‘Y’ (Figure 2). The non-ferromagnetic properties of the titanium material of the head-post prevented the very unlikely (brief or lasting) magnetization and heating of these elements under the influence of isolated TMS single pulses.

### Saccade Behavioral Training and TMS Familiarization

Prior to the first experimental session, animals were specifically trained in an antisaccade (AS) paradigm, in which they were required to initially keep fixation on a red central stimulus. Between 200–700 milliseconds after fixation onset, simultaneously to the disappearance of the central fixation stimulus (no gap), a green square appeared for 1000 ms at a 16° right or left location. Monkeys were trained not to look at this peripheral target but instead, initiate as soon as possible a saccade towards the opposite direction (Figure 1, upper panel). After the saccade, the monkey received a reward if the saccade fell within a 5°×5° window centered at the mirror location of the visual target. Failure to trigger a saccade within 2000 msec after target onset cancelled the trial.

The AS task was chosen since prior human TMS experiments have revealed prosaccade paradigms to be much less sensitive to single pulse TMS interference than antisaccades [9].

Over 4–6 weeks prior to the first experiment, animals underwent specific training to get used to the clicking noise and the scalp tapping sensation accompanying the delivery of TMS pulses, which initially made them blink systematically, even as the coil remained away from their scalp. During the training process, animals were chaired, head-posted and then exposed to sham TMS pulses, by placing an active coil 10–15 cm away from the scalp in different locations around their heads (next to the front, eye canthi, top of the scalp and ears), while performing the AS task and being rewarded for correctly performed trials. Under similar conditions, monkeys were then accustomed to active TMS pulses delivered at progressively closer distances from their left and right frontal hemiscalp, around the location of the FEF, and ultimately directly on both the right and the left FEF sites. New training series followed with the presence of a TMS pulse randomized across trials to avoid predictability and limit biases caused by anticipatory saccade responses. Such TMS familiarization regime dramatically reduced blinking in both monkeys and made animals more at ease, performing the task under the impact of online TMS stimulation.

Eye movements were recorded with an infra-red eye tracker (ASL, Applied Science Laboratories, USA), and eye position was digitized and sampled at 240 Hz and stored for off-line analyses. Visual paradigms and data acquisition were under the control of a computer running a real-time data acquisition system (REX software; for further details see, [42]). Saccades were detected using a computer algorithm that searched first for significantly elevated velocity (>30°/s). Saccade initiation and termination were then defined as the beginning and end of the monotonic change in eye position lasting 12 ms before and after the high-velocity gaze shift. On the basis of the 250-Hz sampling rate, this method is accurate to within 4 ms.

### TMS Stimulation Method and Site

During experiments, TMS was delivered by means of the smallest of the commercially available stimulation tools, a custom-made ∼25 mm radius figure-of-eight TMS coil, normally used for human peripheral nerve stimulation (MagstimCompany, Carmathenshire, Wales). The coil was attached to a single pulse monophasic TMS machine (monopulse, Magstim Company, Carmathenshire, Wales). This same choice of equipment proved to provide efficient motor cortex stimulation in a 5 and a 7 year old rhesus *maccaca mulata* monkeys, that in a recent study showed motor thresholds around 25–30% of an identical monophasic TMS machine maximal output [38].

**Figure 3:**
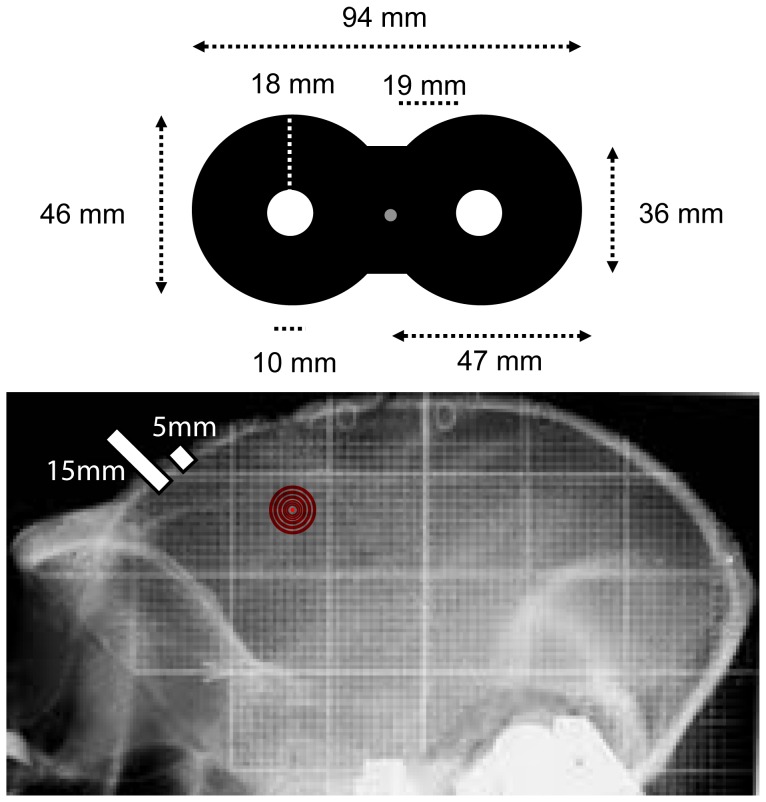
TMS coil positioning. Schematic drawing of the smallest of the commercially available coils, which was used for this experiment (*Upper panel*), a custom-made ∼25 mm loop radius figure-of-eight TMS coil (exact dimensions of the coil used indicated in the figure) (Magstim Company, Carmathenshire, Wales). (*Bottom panel)* X-rays photography of monkey ‘C’. The red target represents the estimated stereotaxic coordinates of the monkey’s FEF area. The length of the white bar illustrates approximate differences in bone thickness between the human and the macaque skull at frontal locations.

The FEF field was identified according to stereotaxic coordinates for this location and its site labeled with a color marker on the monkey scalp, which lasted for several weeks and was renewed when fading. The figure of eight TMS coil center was positioned over this location at every session and oriented in a lateral-to-medial and caudal-to-rostral 45-degree orientation with regards to the scalp midline. The TMS coil was held steady on the same position by means of a well-tightened 180-degrees-of-freedom short mechanical arm attached to the upper lateral side of the monkey chair, ending in a C rubber clamp (Figure 2 and 3). The monkey head was posted to avoid any movements. The TMS coil pulse can generate some brief skull vibration (the monophasic pulse may last ∼120–150 microseconds) but such effects are minimized if not non-existent with single pulse paradigms as the ones we used in this study. In any case, we visually inspected at the beginning and the end of each block the position of the coil on the targeted area to make sure its position did not shift with regards to its target.

Prior to the first FEF stimulation session, the cortical hotspot for the APB (*Abductor Pollicis Brevis*) muscle and the approximate motor threshold (MT) for each animal were determined, as the TMS intensity inducing 50% of the times (out of 10 stimulation attempts) thumb twitching responses. The MT values for the two monkeys, ‘Y and ‘C’ proved slightly higher than those described by Amaya et al. (Monkey ‘Y’: Right M1 45% and Left M1 40%; and Monkey ‘C’: Right M1 40% and Left M1 38% of maximal TMS machine output). Then, during TMS sessions, the center of the TMS double coil site was placed on an area above the expected location of the left (monkey ‘Y’) or the right FEF (monkey ‘C’) (Figure 2). The monkey and human neurostimulation literature seem to consider the left and right FEF as rather mirror symmetric (non lateralized structures) with regards to the control of saccadic activity. We thus hypothesized that the stimulation of either FEF (either left or right) would show mirror symmetric effects, and would result in identical but side-inverted patterns of antisaccadic effects in the two animals.

For this study, we did not have access to a monkey neuronavigation system, neither MRI scans for these two animals, that were implanted with non-ferromagnetic titanium head posts, that generate broad shadowing artifacts in MRI images. Hence, the FEF site was identified by means of stereotaxic coordinates classically used to locate the electrophysiological recording chamber (i.e.,+24 mm;+17 mm, respectively from 0; 0 ear bar position) and labeled on the animal’s scalp. The position for the FEF was further verified as being slightly more rostral (∼1.5–2 cm) and medial (1.0–1.5 cm) than the scalp hotspot from which we could optimally elicit TMS evoked hand muscle twitching (Figures 2 and 3) in both monkey. To assess the effect of intensity, active or sham TMS pulses at three different intensities were tested within the same session, low (30%), medium (40%) and high (50%), at least in three sessions. In the two monkeys of this study, ‘C’ and ‘Y’, these stimulation levels corresponded respectively, to 75% and 79%, (low), 100% and 105% (medium) and 125% and 131% (high) of the individual motor threshold determined in the M1 area of the targeted hemisphere in each animal. No signs of hand twitching for any of such three intensities tested were evoked or sensed by palpation in the upper limb during FEF TMS stimulation.

The effects of TMS intensity, TMS pulse timing and TMS modality or site were evaluated in separate experimental sessions (see Figures 2 and 3). TMS pulses at each of those three levels of intensity were also delivered in separate sessions at an early (100 ms) and a late (150 ms) stimulus onset asynchrony interval (SOA), post visual target appearance, which was selected based on preliminary monkey’s saccade latency measurements. In order to keep conditions as similar as possible, during sham TMS sessions the coil was first placed above the expected location for FEF region and then moved up 4 cm above the skin, keeping an identical orientation. Under such conditions the magnetic field was separated away from the area of interest, providing an acceptable control for the clicking sound of the coil at the two SOAs employed in the study. This type of control is commonly used in human TMS experiments and it mimics but does not perfectly match every type of sensory stimulation linked to the delivery of TMS single pulses. This is in part due to the fact that the small TMS coil used in our experiments needed to be cautiously separated from the scalp to avoid the magnetic field to exert a meaningful influence on the underlying cortex. For further verification, and to overcome some of the limitations of the latter control, animals were also actively stimulated in a dorsal control location within the same hemisphere, adjacent (but not in direct contact) to the lateral and caudal portion of the head post platform. It consisted in the delivery of real TMS pulses on an area of the parietal skull underlying a rostro-dorsal parietal location, at the same TMS intensity. This active condition, which is also commonly used in human TMS experiments, provided an adequate control for every sensory accompanying sensation the stimulation might produce. This condition was carried over once at 150 ms post target SOA, which was the TMS pulse timing that yielded the most robust AS neuromodulatory effects in both animals. This condition was carried over once at 150 ms post target SOA, which was the TMS pulse timing that yielded the most robust AS neuromodulatory effects in both animals.

Neither the FEF nor the active control site (located adjacent to the lateral and caudal aspect of the head-post base, but not in contact with it) were located beneath the titanium head post of directly under one of its titanium “X" shaped plate attachments. In other words, the center of the 25 mm figure of eight coil remained for all conditions in direct contact with the skin underlying cranial bone (whether mid frontal or dorsal parietal) overlying those locations and away from the contact of any of the head post elements.

Overall, in every session, active or sham TMS pulses were randomly delivered only in 50% of the trials to avoid any pulse predictability. The inter-trial interval, i.e., the time lapse between two TMS pulses or saccades was kept ∼4 seconds to avoid unlikely carry-over effects within the session.

### Session and Study Organization

In a series of independent experiments, animals performed a total of 4 blocks of AS training per session. In one of the blocks animals received no TMS at all, whereas in the remaining 3 blocks, they were actively stimulated at each of the 3 different TMS intensities mentioned above (30%, 40% and 50% of maximal machine output). The order of the four blocks within a session was randomized and each session (testing a given region, FEF or control, SOA, 150 or 100 ms, and TMS modality, active or sham) was repeated up to three times (#1, #2 and #3). Monkeys performed 100 trials per block for a total of 400 trials per session, and received 50 pulses per TMS block, and a maximum of 150 pulses per testing session (Figure 1, bottom panel). As indicate above, animals underwent also identical sessions with sham TMS stimulation and active TMS stimulation on a control area at the same three TMS intensities and SOAs described above. Overall (see Supplementary Table S1 for detailed results), each of the two monkey performed 3 sessions of active FEF TMS delivered 100 ms post-target onset, 3 sessions of active FEF TMS delivered 150 ms post-target onset, 2 sessions of sham FEF TMS delivered 150 ms post-target onset and 1 session of active/control TMS delivered 150 ms post-target onset. Sessions of active and sham TMS were interleaved, so that active stimulation sessions were always separated at least 72 hours or longer to avoid very unlikely inter-session carry over effects.

### Data Analysis and Presentation

**Figure 4:**
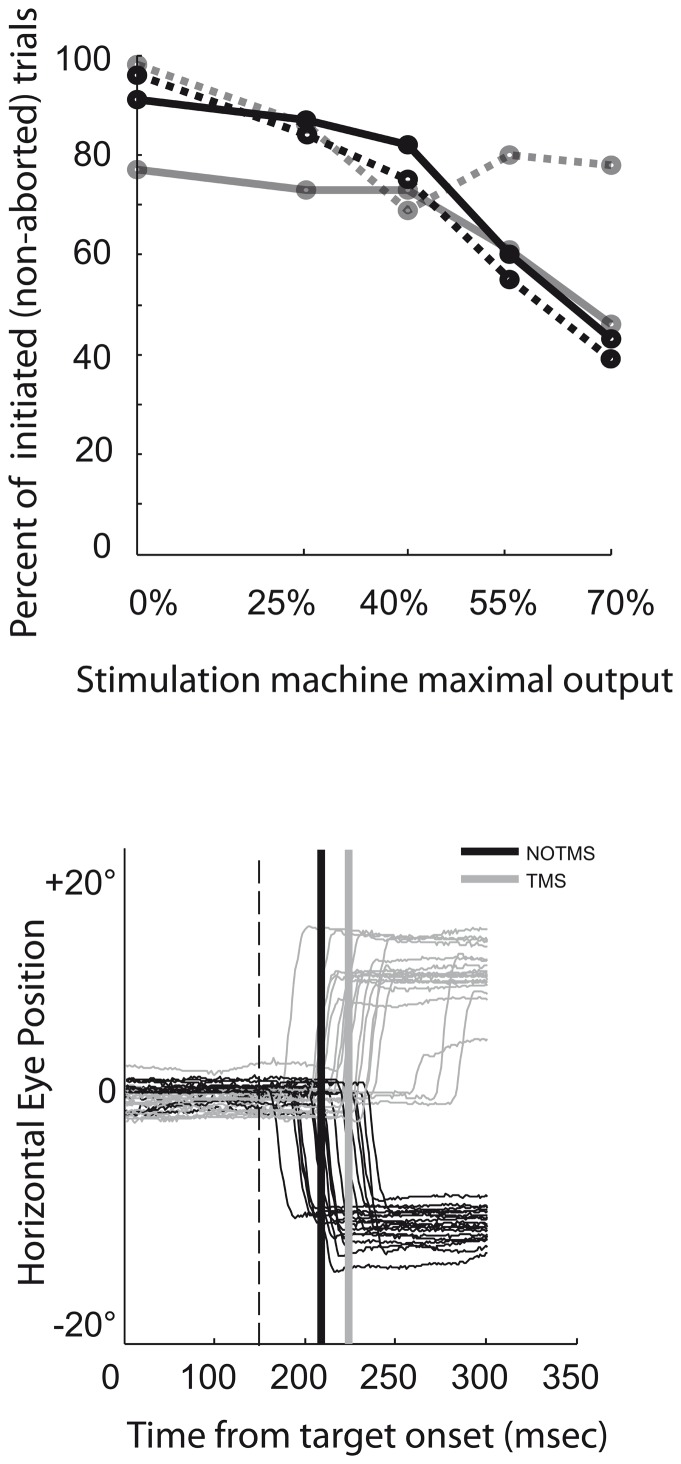
Estimate of discomfort induced by TMS. Estimation of discomfort based on the interaction between percent of initiated trials (used as an indirect correlate of the level of discomfort; the higher the discomfort the lower the number of initiated trials) and TMS intensity (% of machine maximal output). (*Upper panel)* Note that below 50% intensity, low discomfort is inferred from the high percent of initiated trials (grey and black lines) respectively for monkey ‘C’ and ‘Y’, for the TMS condition (dotted lines) as compared to the sham TMS condition (solid lines). (*Bottom panel*) Representative traces of eye movement with/without TMS (respectively grey, black lines) at 50% intensity for one of the monkeys. At least for the two SOAs used in the study (100 and 150 ms) eye movement metrics were not affected by TMS. Furthermore, no saccades were elicited by the stimulation.

Trials with blinking responses interfering with eye recordings or incomplete AS were eliminated from the data set. Such trials represented less than 2% in the No TMS condition, 5% for low level and 20% in high TMS stimulation, respectively (see Figure 4). The AS latency for each individual trial was calculated as the time between stimulus presentation and the onset derivative of the eye saccade velocity reaching a speed of 30 deg/s. Individual AS latency values were averaged for trials under TMS (TMS-trials) and compared to those without stimulation (no-TMS trials), for each session and experimental condition explored in the study on each monkey (see Supplementary Table S1 for average antisaccade raw latency values for each monkey and experimental condition in milliseconds). Sets of saccade latencies under active/sham and no-TMS trials for each experimental condition were statistically compared by means of non-parametric signed Rank-Sum Wilcoxon tests.

**Figure 5:**
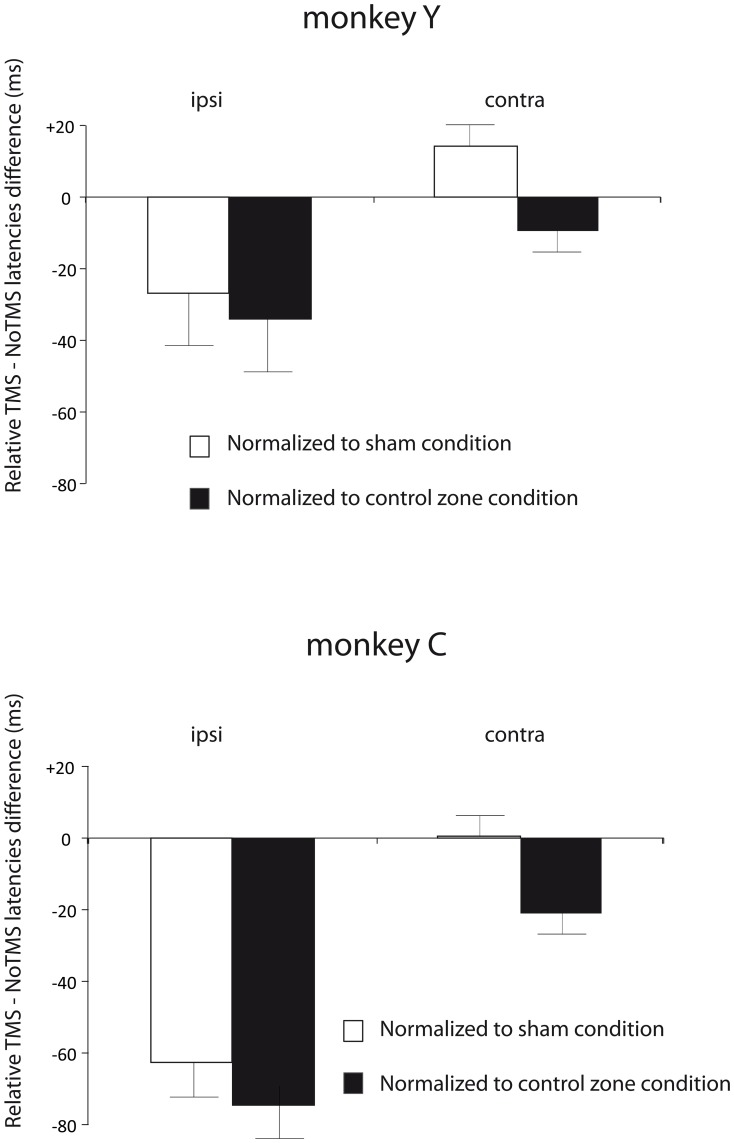
Saccade latencies in TMS or no-TMS trials. Relative modulation of antisaccades latencies (mean and SD) under the impact of online FEF TMS normalized by the effects of sham TMS (white columns; (real TMS-noTMS)-(sham TMS-noTMS)) on the FEF, or active stimulation on a control cortical site (black columns; (real TMS-noTMS)-(active control TMS-noTMS)). Data are shown in millisecond differences for each of the two monkeys (‘C’ and ‘Y’) with TMS delivered at a SOA of 150 ms pre-target onset and at high intensity (50%), at which the effects of active FEF TMS were mostly noted in both monkeys (see Supplementary Table S1 for details). Decreases in normalized AS latency differences suggest a TMS-induced acceleration of AS preparation time with regards to the observed effects for sham TMS (white columns) or active TMS (black columns) in a control site and vice-versa. Notice that in both animals (‘C’ and ‘Y’) active TMS pulses decreased the average latency differences of the AS towards the hemifield ipsilateral to the stimulated FEF, whereas changes were marginal or null for contralateral AS.

In order to control for the potential lateralized biases generated by TMS accompanying sensory phenomena, we normalized by subtraction the AS latency modulations induced by active TMS at the 150 ms SOA by those observed in homologue experiments under sham TMS ((real TMS-noTMS)-(sham TMS-noTMS)) or active TMS on a control cortical area ((real TMS-noTMS)-(active control TMS-noTMS)). This allowed us to eliminate inter-session variability, with regards to baseline AS latency value and to better define the characteristics (direction and field specific effects) of such modulation in each of the two monkeys. Indeed, whereas the raw AS latency effects were not always fully consistent across the two monkeys (see Supplementary Table S1), data showed inter-individual consistence only when such normalized values were employed (see Figure 5).

Decreases in normalized AS latency differences suggest a TMS-induced acceleration of AS preparation time with regards to the observed effects for sham TMS (white columns) or active TMS in a control site (black columns) and vice-versa. Values around 0% normalized values suggest lack of added effect on AS latency of real TMS with regards to the sham or active control conditions (see Figure 5, for details). Sets of normalized antisaccade latencies towards the hemifield ipsilateral or contralateral to the stimulated FEF were also statistically compared by means of non-parametric signed Rank-Sum Wilcoxon test. The results of this normalization procedure, even when popularly used in TMS human experiments, needs to be interpreted carefully, and always in light of the raw antisaccade latency data presented in see Supplementary Table S1 of this manuscript.

## Results

### Effects of TMS on Animal Comfort

To estimate the degree of discomfort experienced by the animal during the TMS sessions, we calculated the proportion of trials that were voluntarily initiated by fixating the central red cue, and shortly later, aborted by producing a saccade away from central fixation before target presentation. Animals that avoided TMS stimulation lost access to juice reward, and a series of consecutive voluntarily aborted trials, could result in long periods without access to positive reinforcement. During baseline recordings, in absence of TMS such behavior was rarely observed. However, under active or sham TMS, the percentage of initiated and successfully completed trials decreased (Figure 4, upper panel). During active TMS sessions at 40% intensity (i.e., ∼100% of their individual motor threshold), both monkeys initiated and completed just slightly less trials than at baseline, when TMS was delivered at 0% intensity. Nonetheless, at 70% absolute TMS intensity (i.e., at 170 and 184% of each animal’s motor threshold, respectively) both monkeys initiated only 40% of the trials. On the basis of those comfort-related measures, TMS intensity was never increased above 50% of the maximal TMS machine output during the subsequent experiments.

### Effects of Sham and Active TMS over a Control Cortical Site

We gathered evidence on the TMS magnetic pulse-dependent specificity by applying sham TMS stimulation on the FEF at both 100 and 150 ms SOA. Saccade latencies for ipsilateral or contralateral AS did not vary under stimulation at either SOA post target onset or monkeys for any of the three TMS intensities, 30, 40 or 50% (p>0.1, for both monkey ‘Y’ and ‘C’). The location specificity of the stimulatory FEF effect delivered at the 150 ms SOAs was controlled by stimulating at the same intensity and SOA, a cortical control site bearing no direct relation with the processing of AS. No significant AS latency modulations were found under high intensity TMS at 30, 40 or 50% delivered in such control location (*p*>0.1 for both monkeys ‘Y’ and ’C’).

### Effects of Active TMS over FEF 100ms after Target Onset

As shown in Supplementary Table S1, ipsilateral and contralateral (with respect to the TMS stimulated FEF) mean AS latencies were rarely affected by high intensity pulses of active TMS, as compared to the no-TMS condition, when applied 100 ms after target onset. More specifically, neither 30% (low) nor 40% (medium) intensity pulses yield any significantly different mean AS latencies independently of the side (*p*>0.1). However, 50% (high) intensity TMS pulses occasionally induced (only in 1 of the 3 sessions) average latencies, which for ipsilateral AS were significantly shorter than those of the no-TMS condition in both animals (respectively, 222 vs. 265 ms and 229 vs. 272 ms for monkey ‘Y’ and monkey ‘C’ respectively; *p*<0.025).

### Effects of Active TMS over FEF 150 ms after Target Onset

TMS stimulation delivered 150 ms after target onset did affect antisaccade latencies in an intensity dependent manner. At 30% (low) intensity, no significant differences were observed neither for contralateral nor ipsilateral saccades (*p*>0.1), in any of the two monkeys. Following active TMS at 40% (medium) intensity, mean antisaccade latencies appeared significantly increased for the contralateral AS in two of the testing sessions performed by monkey ‘Y’, and shortened in only one sessions for monkey ‘C’ ipsilateral antisaccades. Finally, following 50% (high) intensity TMS pulses applied 150 ms after target onset, mean saccade latency was longer for contralateral saccades for monkey ‘Y’ and shorter for ipsilateral saccades in monkey ‘C’ (see Supplementary Table S1).

In order to rule out the influence of potential lateralization biases induced by the clicking noise and scalp tapping sensations linked to the stimulation, we normalized by subtraction the modulations induced by FEF TMS by the effects of sham FEF TMS and active TMS in a control cortical location, both derived from interleaved experimental sessions. According to this analysis (Figure 5), our data indicate that in both monkeys, FEF TMS induced relative decreases in AS latency towards the visual hemifield ipsilateral to the stimulated FEF (vs. Sham condition: -27 ms and -63 ms; vs. active control TMS site: -43 ms -75 ms; *p*<0.025, respectively for monkeys ‘Y’ and ‘C’). In contrast, it interfered very little AS activity directed to the opposite hemifield (vs. Sham condition: +14 and 0 ms; vs. active control TMS site:+19 and -21 ms; *p*>0.5, respectively for monkeys ‘Y’ and ‘C’).

### TMS Effect on Antisaccade Error Rate and Amplitude

The impact of TMS stimulation over FEF on AS error rate was statistically tested separately for each TMS condition site. As compared to sham TMS or active TMS on the control location, stimulation over the FEF did not appear to significantly affect error rate in any of the experimental conditions tested (*p*>0.3, for all conditions in both animals). It should also be noted that at least for the two SOAs used in the study (100 ms and 150 ms) eye movement metrics were not affected by the TMS pulses. More specifically the difference of saccade amplitude between TMS and No-TMS condition was negligible (Monkey C: 0.28 and 0.52 degrees of visual angle; Monkey Y: 0.15 and 0.22 degree of visual angle also for ipsilateral and contralateral antisaccades with regards to the stimulated FEF, respectively). Furthermore, no saccades were elicited by the stimulation (Figure 4, bottom panel). Finally, as compared to sham TMS or TMS active stimulation in a control region, active stimulation over the FEF did not significantly affect AS amplitude in any of the experimental conditions (*p*>0.5, for all conditions in both animals).

## Discussion

Our study demonstrates that single pulse TMS delivered on the FEF region induces a robust modulation of AS preparation latency in the awake behaving monkey. Such effect proved to be specifically induced by the magnetic field on such stimulated frontal site, since no significant modulatory effects were observed neither during sham TMS of the same area, nor when active TMS pulses at identical intensities and timings were delivered over a cortical control region. The interference proved also intensity specific, being null at subthreshold levels (∼75% of motor threshold), weak and occasional with TMS at motor threshold levels and solid and reproducible at the highest TMS intensity that animals tolerated well (∼130% of motor threshold).

To the best of our knowledge, this is the first published report in which TMS is used on the awake behaving monkey to demonstrate *online* interferences on a cognitive function; more specifically on the planning of antisaccades by the FEF. As popularly done in human cognitive applications, our study attempted a trial-by-trial interference of FEF contribution to time-locked processes with bearing on the planning of visually guided eye movements. Accordingly, we targeted in monkeys, the FEF, which can be considered a homologue site to those often studied, in human TMS saccadic experiments. Stimulation was applied using an identical procedure, and delivered at equivalent intensities as those normally used in humans. Furthermore, its effects were recorded as shifts in the normal processing time for such computations as also done in human FEF mapping explorations [6], [7], [8], [9], [15], [16].

It is well known that stimulation generates a clicking noise and provides a light scalp tapping sensation, which is commensurate to stimulus intensity. Those effects are not painful, but can be surprising, distracting and eventually bothering to both, humans and animals. Our study proves that after a relatively short familiarization period, monkeys were able to tolerate *online* TMS, which had to be however kept carefully within a specific range of intensities. Furthermore, animals could bear with the TMS accompanying side-effects, while performing an antisaccadic task without major distress. A familiarization training reduced the frequency of TMS pulse-associated blinking, which were present initially, even under sham stimulation (thus likely to be induced by the loud sound generated by the TMS coil). We used an indirect quantitative parameter, such as the number of aborted trials, as an indicator of animal’s self-tolerability and comfort. Our scale might appear difficult to compare to other scores that have been validated in humans using subjective reports of discomfort. However, measurements seemed to reflect a reasonable estimate of discomfort for the animal, as annoying sensations decreased with practice and occurred much less frequently, when TMS stimulation was kept no higher than 50% absolute intensity (∼130% of each animal’s motor threshold).

We focused our work on the evaluation of AS rather than prosaccades, because prior research suggested very limited on line and offline modulations of the former with TMS single pulses or patterns [9], [39]. Taken as a whole, our results proved coherent with human TMS saccadic studies. As expected, for both monkeys, only TMS on the FEF site at a significant level of intensity (∼50%), but not below, consistently modified the duration of antisaccade latencies. Similar interventions on the human frontal or parietal cortices led to significant changes in either prosaccades [6], [7], [8], [43] or antisaccades preparation time [9], [10], [12]. The lack of effects on AS errors and amplitudes observed in our data is also coherent with prior FEF lesion studies [44], [45] and also TMS explorations of the intact frontal cortex in healthy humans [9], [10]. A more detailed analysis of raw saccade latency data revealed however somehow diverse patterns of modulation, with significantly faster ipsilateral AS in the monkey stimulated on the left FEF and slower contralateral AS in the animal stimulated on the right FEF. Such discrepancies could had been caused by side specific and individual sensory biases generated by the TMS associated clicking and tapping scalp sensations, and thus called at least for a normalization of the raw AS latency data by the impact of sham FEF TMS or active stimulation in a control area, in which similar sensory sensations were present. Interestingly, such new analyses reflected for both monkeys a common pattern of effects showing consistent latency decreases for AS directed towards the hemifield ipsilateral to the TMS stimulation in response to a contralateral visual signal. Nonetheless, this normalization procedure, even if it is popularly used in TMS human experiments and results in a more consistent pattern of effects across the two evaluated animals, provides additional weight to the influence of TMS unspecific effects on monkey saccadic behavior, and thus needs to be interpreted carefully, and always in light of the raw data presented in Supplementary Table S1.

Differences of FEF TMS for ipsilateral and contralateral saccades are not surprising and have been reported in previous human TMS antisaccades studies, which showed active modulation of rightwards but not leftwards AS with single TMS pulses delivered post target onset on the right FEF [9]. For this study, we did not have access to a monkey neuronavigation system, neither MRI scans for these two animals, which were implanted early on with non-ferromagnetic head posts that generate broad artifacts in MRI images. We thus, determined the FEF localization according to stereotaxic coordinates normally used for the implantation of recording chambers prior to a craniotomy. Nonetheless, supporting the known precision of a stereotaxically based FEF localization procedure, our TMS intervention generated saccadic modifications that are compatible with the interference of FEF activity. Furthermore, given, the spatial resolution of TMS, estimated between 1.2–1.5 cm radius [29], [46], the TMS effects should remain relatively invariant to small targeting errors.

At difference with respect to the current results however, human TMS saccadic literature has generally reported net relative increases in prosaccades and AS latencies after FEF stimulation, rather than the decreases revealed by our normalized data in monkeys. Hence, in light of such results, our observations would not be compatible with the inhibition of pro-saccadic mechanisms necessary to complete an AS, but with a potential suppressive effect of single pulse TMS on FEF fixation neurons, which could accelerate the conclusion of an AS by reaching a location opposite to the peripheral visual signal. The selective effects shown by TMS on such subpopulation of FEF neurons could have been facilitated by the relative spatial segregation of saccadic and fixation neuronal populations in the monkey FEF and the lower threshold of the latter as compared to the former [18], [47], [48], and is in agreement with a similar finding reported by a recent offline TMS study on pro-saccades [39]. Even if those studies used at first view different TMS approaches to the interference of cortical activity and cognitive processing, both neurostimulatory patterns (*offline* continuous theta burst stimulation in [39] and *online* single pulse stimulation in the current study) are known to induce respectively lasting and brief suppressive effects on cortical activity, and thus their effects can to some extend carefully compared. Unfortunately, in absence of further monkey TMS studies in which the effects of magnetically induced current can be characterized on different populations of FEF neurons located at different regions and depths, such explanation remains purely speculative.

On the basis of our interventions, we cannot exclude that regions other than the targeted FEF, located at a reasonable distance from this site, could have also been collaterally impacted by the stimulation. Consequently, the current TMS effects on antisaccades should be considered rather preliminary. However, It should be argued in our favor, that at the expense of a lower penetration power, we used the most focal TMS coil currently commercially available (25 mm figure of eight coil); we worked at rather low TMS intensity levels (30 to 50% of maximal TMS machine output), fact that should have limited radial spread; and we employed isolated TMS pulses, which is the mildest, most precise and shortest lasting TMS stimulation paradigm available. In sum, given the currently available TMS equipment and authorized non-invasive procedures, we did our best to minimize magnetic field radial diffusion and favor the highest spatially selective effect possible. Moreover, even if the accepted spatial resolution of TMS has been estimated between 1.2 and 1.5 cm, this could be considered a pessimistic estimation determined with TMS coil sizes, patterns and stimulation intensities [29], [33], [46], [49] non-necessarily comparable to those used in our study. Indeed, several pieces of evidence suggest that the spatial resolution delivering single TMS pulses with a 25 mm figure-of-eight coil as ours, rarely used in brain stimulation, might prove in the monkey lower than the estimations indicated above. First, Gerits et al. [39], recently claimed using this same TMS coil in the adult male rhesus monkey, highly focal offline saccadic effects after the delivery of prolonged continuous theta burst TMS patterns. Second, Amaya et al [38] used the same coil to map with single suprathreshold pulses also in the rhesus monkey, the localization of several motor representations, with an estimated final spatial resolution of 0.5 to 1.0 cm; Finally, the diffusion of the magnetic field operates radially [29], [33], and hence the same way it could have significantly influenced the rostrally located prefrontal or premotor cortex, it should have also caudally impacted the primary motor cortex of our animals, located at a similar distance from the FEF; Nonetheless, we did not observe any sign of motor activation generated by magnetic field diffusion for any of the intensities used during our experimental sessions. All this evidence taken together, suggest that the single TMS pulses used in our experiment are likely to have selectively acted on the targeted area and thus had only minimally influenced nearby sites.

In general terms, the found neuromodulatory effects could be considered consistent with the classical effects of *online* TMS as a procedure to transcranially transport electrical charge and induce intracortical currents which might contribute “noise" to the communication effort developed by local FEF networks involved in fixation, during their attempt to generate appropriate behavior [33]. Another potential mechanism could consist in the induction of lasting hyper-polarization by some alterations in extrinsic synaptic input or intrinsic membrane properties of intracortical FEF interneurons. Similarly, electrical microstimulation has been shown to substantially elevate the levels of extracellular cortical GABA, an effect that has the ability to suppress firing activity [50]. Thus, neuronal suppression may result from the disruption of normally coordinated activity patterns at the circuit level. In support of this idea, very recent work has noted the key role played by local GABAergic microcircuitry to explain suppressive local and distant modulation phenomena [51]. Finally, several lines of evidence have shown that, the temporal relationship of neural signals, as measured by spike-LFP and LFP-LFP phase synchrony in animals [30] and humans [52] are selectively altered following TMS stimulation, and might indeed ultimately explain brief or lasting local and distant cognitive interference. Although we have no means yet to rule out any of these potential mechanisms, an awake behaving monkey model of *online* TMS effects would be perfectly suited to put some of those hypotheses to test in a near future.

In the last decade, non-invasive neurostimulation has provided terrific insights into the causal mechanisms of cognitive processes, and has also contributed some interesting beneficial effects for neurological and neuropsychiatric patients [33]. Moreover, non-invasive brain stimulation devices remain a top interest to develop safe neuroprosthetics and therapeutically efficient and safe neurostimulation devices. Intracortical microstimulation as performed in monkeys can claim selective stimulation of different FEF and overlapped sub networks by considering the precise anatomical location of such systems, their retinotopic and receptive field organization or the different excitability thresholds shown by the neural populations located within [3], [4], [18], [53], [54], [55]. Regardless of the great advantage provided by its non- invasiveness, TMS will always badly compete with microstimulation in terms of precision and spatial resolution. Nonetheless, further insights on the TMS mechanisms of action gathered through animal models such as the one presented in this study, might allow a more precise targeting of neural populations based on stimulation intensity and coil positioning. Furthermore, a combination of poorly focal non-invasive neurostimulation with cortical adaptation paradigms could allow us to differentially modulate the activity state of overlapped FEF systems, and prime the TMS effects on more selected neuronal subnetworks (Silvanto and Muggleton, 2007).

Thus far, we presented evidence that the synchronized *online* TMS modulation of a saccade related processing is feasible in the awake behaving macaque monkey. Our data support hopes that monkey TMS holds the potential to become a well-suited model to enhance our understanding of brain function and non-invasive neurostimulation mechanisms, in particular if combined with interleaved local field and single unit recordings with implanted intracerebral electrodes. It is only on the base of such contributions that neurostimulatory approaches will be optimized to provide efficient neuromodulatory therapies for human patients.

## Supporting Information

Table S1 Mean ipsilateral or contralateral AS latency (in ms) and standard error of the mean for each of the two monkeys tested (‘Y’ and ‘C’), under active or sham TMS at each of the 3 TMS intensity levels (low, 30%, medium 40%, and high 50%) and target onset timing (100 or 150 ms) of our study. Data for the corresponding no-TMS blocks carried out at each session (#1, #2 and #3) is also displayed. Session #2 data for 100 ms SOA and medium TMS intensity for monkey C is missing because of a technical problem during the recording session. Statistically significant values * p<0.05, ** p<0.01 or *** p<0.001 with respect to the equivalent noTMS AS latencies measured within the same session have been signaled in bold. Statistically marginal significant effects (p = or<0.07) have been explicitly indicated in the table. The text “ns" indicates non-statistically significant effects of a given TMS condition with respect to its no-TMS same session counterpart. Monkey ‘Y’ (upper table) displayed under medium and high intensity left FEF TMS consistent and statistically significant latency for ipsilateral AS in a majority or the tested sessions, mainly at 150 ms SOA. Under high intensity right FEF TMS Monkey ‘C’ (lower table), showed consistent statistically significant accelerations of ipsilateral AS mainly for the 150 ms SOA, and at each of the three sessions tested. Neither low TMS intensity nor sham TMS or active TMS on a control location resulted in statistically significant modulations of AS latencies. See Figure 5 for a representation of the AS latency modulation data normalized by the effects of Sham TMS or active TMS in a control location.
(PDF)
