# Supplementary material for: Frontal Non-Invasive Neurostimulation Modulates Antisaccade Preparation in Non-Human Primates
Source: PLoS One. 2012 Jun 6;7(6):e38674. doi: 10.1371/journal.pone.0038674 (PMC3368878; doi:10.1371/journal.pone.0038674)
Supplement: Table S1 — Mean ipsilateral or contralateral AS latency (in ms) and standard error of the mean for each of the two monkeys tested (‘Y’ and ‘C’), under active or sham TMS at each of the 3 TMS intensity levels (low, 30%, medium 40%, and high 50%) and target onset timing (100 or 150 ms) of our study. Data for the corresponding no-TMS blocks carried out at each session (#1, #2 and #3) is also displayed. Session #2 data for 100 ms SOA and medium TMS intensity for monkey C is missing because of a technical problem during the recording session. Statistically significant values * p<0.05, ** p<0.01 or *** p<0.001 with respect to the equivalent noTMS AS latencies measured within the same session have been signaled in bold. Statistically marginal significant effects (p = or<0.07) have been explicitly indicated in the table. The text “ns" indicates non-statistically significant effects of a given TMS condition with respect to its no-TMS same session counterpart. Monkey ‘Y’ (upper table) displayed under medium and high intensity left FEF TMS consistent and statistically significant latency for ipsilateral AS in a majority or the tested sessions, mainly at 150 ms SOA. Under high intensity right FEF TMS Monkey ‘C’ (lower table), showed consistent statistically significant accelerations of ipsilateral AS mainly for the 150 ms SOA, and at each of the three sessions tested. Neither low TMS intensity nor sham TMS or active TMS on a control location resulted in statistically significant modulations of AS latencies. See Figure 5 for a representation of the AS latency modulation data normalized by the effects of Sham TMS or active TMS in a control location. (PDF) [file pone.0038674.s001.pdf]

Monkey Y

	Low intensity				Medium intensity				High intensity			
	TMS ipsi	TMS contra	NO TMS ipsi	NO TMS contra	TMS ipsi	TMS contra	NO TMS ipsi	NO TMS contra	TMS ipsi	TMS contra	NO TMS ipsi	NO TMS contra
SHAM 100 ms	234 ±36 N.S.	267 ±31 N.S.	199 ±32	264 ±27	227 ±30 N.S.	280 ±32 N.S.	204 ±25	251 ±22	245 ±41 N.S.	302 ±47 N.S.	224 ±31	271 ±31
SHAM 150 ms	227 ±74 N.S.	181 ±113 N.S.	198 ±56	196 ±76	229 ±17 N.S.	365 ±89 N.S.	218 ±19	364 ±90	235 ±38 N.S.	276 ±90 N.S.	223 ±23	275 ±45
Control region 150 ms	210 ±62 N.S.	227 ±24 N.S.	208 ±26	218 ±71	230 ±41 N.S.	223 ±40 N.S.	207 ±25	247 ±33	189 ±50 N.S.	213 ±51 N.S.	160 ±38	184 ±61
FEF 100 ms #1	211 ±112 N.S.	220 ±30 N.S.	195 ±25	230 ±30	244 ±30 N.S.	240 ±25 N.S.	204 ±49	255 ±42	251 ±12 N.S.	248 ±39 N.S.	243 ±38	263 ±61
FEF 100 ms #2	249 ±13 N.S.	245 ±29 N.S.	238 ±15	253 ±18	249 ±12 N.S.	242 ±65 N.S.	246 ±40	241 ±30	251 ±27 N.S.	252 ±14 N.S.	229 ±23	255 ±39
FEF 100 ms #3	269 ±21 N.S.	310 ±130 N.S.	258 ±33	283 ±148	255 ±33 N.S.	245 ±58 N.S.	242 ±55	251 ±37	**222** ±71 **	241 ±56 N.S.	265 ±33	269 ±42
FEF 150 ms #1	216 ±56 N.S.	241 ±29 N.S.	216 ±24	233 ±18	237 ±37 N.S.	279 ±22 N.S.	253 ±40	268 ±95	**216** ±47 N.S.(.07)	**295** ±15 **	254 ±41	253 ±42
FEF 150 ms #2	220 ±32 N.S.	241 ±34 N.S.	225 ±29	244 ±23	212 ±36 N.S.	**276** ±29 *	218 ±27	246 ±19	**285** ±46 N.S.	**295** ±36 **	259 ±28	238 ±74
FEF 150 ms #3	235 ±29 N.S.	257 ±79 N.S.	234 ±29	259 ±99	211 ±47 N.S.	**269** ±35 **	219 ±30	232 ±35	**205** ±7 N.S. (*)	**276** ±55 **	234 ±21	239 ±42

MonkeyC

	Low intensity				Medium intensity				High intensity			
	TMS ipsi	TMS contra	NO TMS ipsi	NO TMS contra	TMS ipsi	TMS contra	NO TMS ipsi	NO TMS contra	TMS ipsi	TMS contra	NO TMS ipsi	NO TMS contra
SHAM 100 ms	224 ±27 N.S.	283 ±49 N.S.	202 ±24	270 ±51	210 ±45 N.S.	253 ±67 N.S.	217 ±42	243 ±38	203 ±46 N.S.	246 ±35 N.S.	204 ±31	236 ±55
SHAM 150 ms	262 ±36 N.S.	194 ±45 N.S.	238 ±21	209 ±37	257 ±48 N.S.	209 ±39 N.S.	253 ±21	203 ±25	183 ±21 N.S.	260 ±52 N.S.	208 ±40	260 ±42
Control region 150 ms	207 ±26 N.S.	211 ±26 N.S.	219 ±45	210 ±32	186 ±50 N.S.	200 ±56 N.S.	205 ±85	198 ±21	184 ±41 N.S.	208 ±28 N.S.	188 ±41	204 ±22
FEF 100 ms #1	263 ±30 N.S.	208 ±49 N.S.	270 ±41	205 ±30	241 ±21 N.S.	222 ±14 N.S.	243 ±28	210 ±24	206 ±56 N.S.	207 ±29 N.S.	263 ±45	211 ±45
FEF 100 ms #2	243 ±66 N.S.	177 ±35 N.S.	287 ±50	201 ±16					**229** ±45 **	**217** ±45 N.S. (0.064)	272 ±72	205 ±24
FEF 100 ms #3	261 ±47 N.S.	206 ±38 N.S.	252 ±53	202 ±12	214 ±34 N.S.	177 ±21 N.S.	207 ±25	180 ±19	254 ±39 N.S.	171 ±24 N.S.	216 ±36	201 ±34
FEF 150 ms #1	270 ±49 N.S.	238 ±27 N.S.	298 ±29	215 ±68	294 ±45 N.S.	231 ±30 N.S.	239 ±49	219 ±28	**204** ±56 **	206 ±45 N.S.	252 ±21	229 ±25
FEF 150 ms #2	269 ±40 N.S.	200 ±44 N.S.	251 ±71	227 ±54	**225** ±33 **	206 ±180 N.S.	262 ±43	224 ±30	**196** ±31 ***	208 ±23 N.S.	254 ±55	234 ±32
FEF 150 ms #3	259 ±45 N.S.	209 ±25 N.S.	260 ±27	212 ±46	225 ±40 N.S.	226 ±38 N.S.	220 ±63	215 ±33	**202** ±35 ***	197 ±11 N.S.	271 ±62	214 ±35

Table S1
